# The epidemiology of headache disorders: a face-to-face interview of participants in HUNT4

**DOI:** 10.1186/s10194-018-0854-2

**Published:** 2018-03-20

**Authors:** Knut Hagen, Anders Nikolai Åsberg, Benjamin L. Uhlig, Erling Tronvik, Eiliv Brenner, Marit Stjern, Grethe Helde, Gøril Bruvik Gravdahl, Trond Sand

**Affiliations:** 1 Department of Neuromedicine and Movement science, Faculty of Medicine and Health Science, Norwegian University of Science and Technology, 7489 Trondheim, Norway; 2 Norwegian Advisory Unit on Headaches, St. Olavs University Hospital, Trondheim, Norway; 3 Department of Neurology and Clinical Neurophysiology, St. Olavs University Hospital, Trondheim, Norway

**Keywords:** Idiopathic stabbing headache, Cluster headache, Migraine, Tension-type headache, General population, Prevalence

## Abstract

**Background:**

The primary aim of this cross-sectional population-based study was to evaluate the 1-year prevalence of common headache disorders by a face-to-face interview.

**Methods:**

The fourth wave of Nord-Trøndelag Health Survey (HUNT4) started in September 2017. The study was undertaken as part of a project mainly focusing on sleep disorders, where a total of 232 (19.3%) out of 1200 invited HUNT4 participants underwent a face-to-face headache interview.

**Results:**

The mean age of the 232 participants was 58.4 years (range 22–89). There were 71.6% (95% CI 65.7–77.4) who reported headache during the last year, and 18.5% (95% CI 13.5–23.6) had suffered from headache in the same period. The 1-year prevalence of tension-type headache (TTH) was 43.1% (95% CI 36.7–49.5), of idiopathic stabbing headache 34.1% (27.9–40.2), and of definite migraine 18.1% (95% CI 13.1–23.1). A total of 7.6% (95% CI 4.0–10.7%) had migraine with coexisting TTH. Lifetime prevalence of migraine was 32.8% (95% CI 26.7–38.8). Headache yesterday was reported by 12.1% (95% CI 7.9–16.3), and 5.6% (95% CI 2.6–8.6) had headache during the interview.

**Conclusion:**

In this population-based cross-sectional headache study performed by a face-to-face interview, the 1-year prevalence of TTH was 43.1% and of idiopathic stabbing headache 34.1%. A total of 18.1% had active migraine (18.1%), whereas the lifetime prevalence of migraine was 32.8%.

## Background

A careful history taken by a headache specialist is the gold-standard for making a valid headache diagnosis. This is, however, time-consuming and costly, therefore relatively few large population-based studies have used a face-to-face interview approach [1–9]. Most large-scale population-based studies have used telephone interview by lay interviewers, a self-administrated questionnaire, or a combination of a screening questionnaire and an interview by a physician [10, 11].

A face-to-face headache interview performed by headache specialist allow distinction between probable and definite headache diagnoses according to the most recent criteria of the International Classification of Headache Disorders (ICHD). Most studies focus on presence of an *active* headache disorder to reduce the influence of recall bias, usually defined as occurrence of headache during the last year [11].

Previously, we have done two face-to-face headache interviews in the general population to validate questionnaire-based diagnosis with diagnosis made by a headache specialist [6, 12]. This is also the overall goal by performing face-to-face interviews, but access to questionnaire-data will first be available during 2019.

The aim of the present study was to estimate the 1-year prevalence of common headache types by using a face-to-face interview in a random sample of participants in a large-scale population-based study.

## Methods

### Study design

This study was undertaken as a part of a large cross-sectional population-based study evaluating the 1-year prevalence of headache disorders by a face-to-face interview.

### The fourth Nord-Trøndelag health survey (HUNT4)

All inhabitants aged 20 years or more in Nord-Trøndelag county of Norway have been or will be invited to participate in the adult version of HUNT4 in the period between September 2017 and March 2019, whereas adolescents aged 13–19 will be invited to Young-HUNT4. Individuals living in the town Stjørdal were consecutively invited to participate in HUNT4 in the period between September 4th, 2017 and February 22th 2018. Individuals living in other study areas will be invited in other periods before March 2019.

### HUNT4 sleep and pain study

The present study is a part of a subproject of HUNT4 mainly focusing on sleep disorders including an invitation to ambulatory polysomnography (PSG) and neurophysiological measurements. A random sample of adults living in Stjørdal who had participated in HUNT4 and answered both the first and the second questionnaire received a written invitation. The invitation letter informed about an initial interview focusing on sleep and pain and to have ambulatory PSG and measurements of pain thresholds performed later. Participants who confirmed to participate received a sleep diary to fill in at home 5–7 days before the interview. Headache was not mentioned in the invitation letter.

### Sample size calculation

We have previously performed a similar still unpublished PSG study as a part of HUNT3. In this study, participants living in Stjørdal were invited to have a PSG in Trondheim. To perform at least 200 PSGs, it was necessary to invite 1200 persons. Thus, based on a participation rate of 17% in this HUNT3 sub-project, we decided to send 1200 postal written invitations randomly to HUNT4 participants. No stratification by sex or age was performed. However, based on previous experience with HUNT2 [12], we knew that the participation would be higher among women than men, highest in the age group 60–69, and lowest in the age group 20–29.

### Headache interview

A semi-structured interview was performed by five medical doctors (three neurologist) with special interest and competence in headache. Initially, all subjects were asked the questions “Have you ever had migraine?”. Those who answered “yes” were asked for age of onset. Later in the interview, we repeated the question regarding age of onset for each type of headache. In the interview, we focused on those who answered “yes” to the question “Have you had a headache during the last 12 months?”.To clarify the burden, we also asked the question “Have you suffered from headache during the last 12 months?” Individuals who reported headache during the past year were asked about frequency (average number of days per month during the last year), time span since last headache, intensity, location, aura symptoms, other migraine and cluster headache features, and use of medication. All diagnoses were based on ICHD-3 version [13]. Up to three different headache types were diagnosed in those with active headache. Subjects with medication overuse headache (MOH) were also categorized according to their primary headache diagnosis.

The participants were also asked the question “Have you during the last year for at least 3 months had pain in muscles and joints?”. Individuals who answered “yes” were defined as having chronic musculoskeletal pain.

The participants also participated in a separate semi-structured interview about sleep disorders with focus on insomnia and restless legs. Individuals were evaluated for insomnia according to DSM-V (Diagnostic and Statistical Manual of Mental Disorders, 5th ed.).

### Ethics

The study was included as a part of the HUNT4 project that was approved by the Regional Committee for Ethics in Medical Research and the Norwegian Data Inspectorate.

### Statistics

1-year prevalence was estimated with 95% confidence interval (CI). The difference between proportions was analysed by chi-square tests, and *p* < 0.05 was considered statistically significant.

## Results

Among the 1200 invited participants, 239 (19.9%) agreed to participate. Among these, seven wanted to participate, but were unable to attend to the interview because they were out of town, had sick husband, were busy at work, or they had forgotten the invitation. Thus, among 1200 invited, 232 persons (19.3%) participated in the headache interview, more women (*n* = 152) than men (*n* = 80). The mean age was 58.4 years (range 22–89), and as demonstrated by Table 1, the majority (70%) of participants were in the age group 50–79 years. A total of 33.2% fulfilled the DSM-V diagnosis of insomnia, and 54.3% suffered from chronic musculoskeletal pain during the last year. The mean number of days between answering the two questionnaires in HUNT4 and the interview was 59 days (range 14–110 days).

**Table 1:** Characteristics of the study population (*n*=232)

	Men	Women
Sex (%)	80 (34.5)	152 (65.5)
Mean age in years (SD)	59.9 (14.5)	57.6 (14.3)
20–29 years (%)	4 (5.0)	6 (3.9)
30–39 years	4 (5.0)	13 (8.6)
40–49 years	8 (10.0)	26 (17.1)
50–59 years	19 (23.8)	32 (21.1)
60–69 years	22 (27.6)	38 (25.0)
70–79 years	19 (23.8)	31 (20.4)
≥80 years	4 (9)	6 (3.9)
Insomnia^a^ (%)	17 (21.3)	60 (39.5)
Chronic musculoskeletal pain^b^ (%)	36 (45.6)	90 (59.6)

^a^Fulfilled the DSM-V (Diagnostic and Statistical Manual of Mental Disorders, 5th ed.) diagnosis of insomnia

^b^Answered “yes” to the question “Have you during the last year for at least 3 months had pain in muscles and joints?”

### Lifetime prevalence of migraine

Lifetime prevalence of migraine was 32.8% (26.7–38.8), 38.8% (95% CI 31.0–46.7) among women and 21.3% (95% CI 12.1–30.4) among men. Mean age of the first migraine attack was 24.3 years (95% CI 21.0–27.5), 24.7 years (95% CI 20.9–28.5) among women and 22.9 years (95% CI 15.8–30.0) among men.

### 1-year prevalence of headache

The 1-year prevalence of different headache disorders by gender among the 232 participants is shown in Table 2. As demonstrated, 71.6% (95% CI 65.7–77.4) reported that they had had headache during the past year, whereas 18.5% (13.5–23.6) stated that they had suffered from headache. The 1-year prevalence of tension-type headache was 43.1% (95% CI 36.7–49.5), 40.1% (336.7–49.5) had episodic TTH and 3.0% (05% CI 0.8–5.2) chronic TTH. The 1-year prevalence of idiopathic stabbing headache was 34.1% (27.9–40.2), whereof 4.7% (95% CI 2.0–7.5) reported attacks with idiopathic stabbing headache at least on a weekly basis. The prevalence of active migraine was 18.1% (95% CI 13.1–23.1), and 7.6% (95% CI 4.0–10.7%) had migraine with coexisting TTH (Table 2). Cervicogenic headache was the most common secondary headache (3.9%, 95% CI 1.4–6.4). Medication overuse headache was present in 0.9%.

**Table 2:** 1-year prevalence of different headache types (*n* = 232). Some participants may have more than one headache diagnosis

	Women	Men	Overall
Number	152	80	232
Idiopathic stabbing headache (%) (95% CI)	41.5 (33.5 to 49.4)	20.0 (11.0 to 29.0)	34.1 (27.9 to 40.2)
Headache, all types (%) (95% CI)	78.3 (71.7 to 84.9)	58.8 (47.7 to 69.8)	71.6 (65.7 to 77.4)
Headache sufferer (%) (95% CI)	23.0 (16.3 to 29.8)	10.0 (3.3 to 16.7)	18.5 (13.5 to 23.6)
Migraine (%) (95% CI) (probable migraine excluded)	23.0 (16.3 to 29.8)	8.8 (2.4 to 15.1)	18.1 (13.1 to 23.1)
Migraine with aura (%) (95% CI)	8.6 (4.1 to 13.1)	3.8 (0.0 to 8.0)	6.9 (3.6 to 10.2)
Migraine without aura (%) (95% CI)	11.8 (6.7 to 17.0)	3.8 (0.0 to 8.0)	9.1 (5.3 to 12.8)
Migraine with or without aura (%) (95% CI)	2.6 (0.1 to 5.2)	1.3 (0.0 to 3.7)	2.2 (0.3 to 4.0)
Migraine with coexisting tension-type headache	9.9 (5.1 to 14.7)	2.5 (0.0 to 6.0)	7.3 (4.0 to 10.7)
Probable migraine without aura (%) (95% CI)	6.6 (2.6 to 10.6)	0.0	4.3 (1.7 to 6.9)
Chronic migraine (%)	0.0	0.0	0.0
Tension-type headache (%) (95% CI) (probable excluded)	47.4 (39.4 to 55.4)	35.0 (24.3 to 45.7)	43.1 (36.7 to 49.5)
Episodic tension-type headache (%) (95% CI)	43.4 (35.5 to 51.4)	33.8 (23.2 to 44.3)	40.1 (33.7 to 46.4)
Infrequent episodic TTH (%) (95% CI)	18.4 (12.2 to 24.7)	18.8 (10.0 to 27.5)	18.5 (13.5 to 23.6)
Frequent TTH (%) (95% CI)	25.0 (18.0 to 32.0)	15.0 (7.0 to 23.0)	21.6 (16.2 to 26.9)
Chronic tension-type headache (%) (95% CI)	3.9 (0.8 to 7.1)	1.2 (0.0 to 3.7)	3.0 (0.8 to 5.2)
Probable tension-type headache (%) (95% CI)	1.3 (0.0 to 3.2)	1.3 (0.0 to 3.7)	1.3 (0.0 to 2.8)
Cervicogenic headache (%) (95% CI)	4.6 (1.2 to 8.0)	2.5 (0.0 to 6.0)	3.9 (1.4 to 6.4)
Medication overuse headache (%) (95% CI)	1.3 (0–3.2)	0.0	0.9 (0–2.1)
Other headaches^a^ (%) (95% CI)	1.3 (0.1 to 3.2)	7.5 (1.6 to 13.4)	3.5 (1.1 to 5.8)

^a^Other headaches (*n* = 8) included; 8.1.4. Alcohol-induced headache (2 cases), headache attributed to 11.5.1 Acute rhinosinusitis (4 cases) or 9.2.2 Systemic viral infection (1 case). One additional case had 8.1.11 Headache attributed to exposure to gasoline using chainsaw

A total of 120 participants were 60 years or older. Comparing to those below 60 years, the 1-year prevalence was lower for migraine (10.0% vs. 26.8%, *p* < 0.001) and TTH (35.8% vs. 50.9%, *p* = 0.024). For cervicogenic headache no significant difference was found (5.8% vs. 1.5%, *p* = 0.17).

### Headache yesterday

Headache yesterday was reported by 12.1% (95% CI 7.9–16.3), 15.1% (95% CI 9.4–20.9) among women and 6.3% among men (95% CI 0.8–11.7), and 5.6% (95% CI 2.6–8.6) reported headache during the interview.

### Impact of being a headache sufferer

Individuals who answered “yes” to the question “Have you suffered from headache during the last 12 months?” were significantly more likely to have active migraine than those who responded “no” (48.8% versus 11.1%, *p* < 0.001).

## Discussion

In this population-based cross-sectional study evaluating 1-year prevalence of headache disorders by a face-to-face interview, TTH was most common (43.1%), followed by idiopathic stabbing headache (34.1%) and migraine (18.1%).

### Comparisons with other studies

Relatively few population-based studies have estimated 1-year prevalence of headache using a face-to-face interview performed by medical doctors with special interest and competence in headache [1–9]. We have previously performed interviews of a random sample of 297 participants of HUNT3, reporting higher 1-year prevalence of TTH (51.9%), but almost similar prevalence of idiopathic stabbing headache (35.0%) and migraine (17.2%) [6]. The 1-year prevalence of migraine of 18.1% in the present study was higher than the 11% and 15.5% reported in the two studies from Copenhagen (1, 4), but lower than found in Italy [7] (Table 3). Furthermore, the 1-year prevalence of 43.1% in TTH was higher than reported in Italy [8], but much lower than reported in the two Copenhagen studies [1, 4]. Interestingly, our 1-year prevalence of idiopathic stabbing headache of 34.1% was identical to the prevalence in Vågå (35.2%) in Norway [3]. As demonstrated by Table 3, our 1-year prevalence of chronic TTH of 3.0% were nearly identical to the corresponding prevalence’s reported in Denmark [1] in Spain [2], and in Akershus in Norway [5]. In contrast, a much lower prevalence of chronic TTH was reported in Italy [8].

**Table 3:** Summary of 1-year prevalence of population-based studies which included a face-to-face interview performed by headache experts

Country (ref)	Published	Age group	Number of participants	Migraine (%)	TTH (%)	CTTH (%)
	(Year)	(Year)				
Denmark [1]	1991	25–64	740	10	74	3
Spain [2]	1999	15+	135	–	–	2.2
Denmark [4]^a^	2005	25–36	207	15.5	86.5	4.8
Norway [5]^b,c^	2008	30–44	633	–	–	2.9
Norway [6]	2008	20–84	297	17.2	51.9	3.7
Italy [7, 8]	2012/2015	18+	904	24.7	19.4	0.6
Egypt [9]^d^	2016	5+	4700	10.5	–	–
Norway	Present study	22–89	232	18.1	43.1	3.0

^a^2001-cohort

^b^Combination of interview performed face-to-face or by telephone

^c^Combination of initial screening questionnaire and personal interview of persons with frequent headache

^d^Neurologist interviewed headache sufferers identified by paramedical staff

In the present study, with a high proportion of elderly participants, cervicogenic headache was common with a total 1-year prevalence of 3.9%. In accordance with this, the lifetime prevalence of cervicogenic headache was found to be 4.1% among 1838 inhabitants in Vågå in Norway [14]. Furthermore, in the present study the lifetime prevalence of migraine was 32.8%. An even higher lifetime prevalence of 40.3% was reported in Vågå [15, 16].

Recent population-based studies initiated by the Global Campaign against headache have focused on headache yesterday, to minimize the influence of recall bias [17]. In the present study, 12.1% reported headache yesterday. Almost similar prevalence was found in Lithuania and Italy [18]. According to summery data from nine European countries, 15–17% had headache yesterday lasting on average for 6 h [18].

The present study did not estimate the 1-year prevalence of very rare headache diagnoses very precisely. In the present study, none of the 232 participants had chronic migraine. In comparison, only two out of 904 participants (0,2%) in Italy fulfilled the criteria of chronic migraine [8]. Such low prevalence may explain why we did not identify participants with chronic migraine. To make relatively precise estimates of rare headaches with prevalence of 0.5% or less, you probably need at least 1500–2000 participants. Merging data from both HUNT3 (*n* = 297) [6] and HUNT4 (*n* = 232), the total sample size of 529 persons is even too small to give precise estimates of rare headaches. Having this in mind, we briefly should mention that we identified one participant with primary exercise headache, indicating a 1-year prevalence of approximately 0.2%. Furthermore, merged data indicated a 1-year prevalence of MOH 1.7%, and a lifetime prevalence of cluster headache of 0.4%. A similar lifetime prevalence of cluster headache was found in Vågå [19].

### Strengths and limitations of the study

The major strength of this study was the population-based design using a face-to-face interview performed by headache experts. The invitation letter did not mention that a detailed headache interview would be performed, hence a selective participation of headache patients is less likely. Several study limitations should also be considered. Since our estimates were based on data from 19.1% of the invited population, one may question to what degree the results can be generalized. The present study mainly focused on sleep disorders, and the very low participation rate could possibly be explained by the supplementary invitation to an ambulatory PSG and neurophysiological measurements. This time-consuming study included traveling to Trondheim two consecutive days, which may not be possible for most full-time workers. Furthermore, most full-time workers could not attend interviews during daytime and we regrettably had no time for afternoon appointments. Thus, the present participants had a high proportion of elderly who could attend, had much more insomnia and slightly more chronic musculoskeletal pain than previously found in HUNT3. In the present study, 33.2% fulfilled the DSM-V diagnosis of insomnia, and 54.3% had chronic musculoskeletal pain. The corresponding figures in HUNT3 were respectively 7.9 and 50.4% [20, 21]. It should be highlighted that a bidirectional association between insomnia and headache has been found, in particular for headache ≥7 days/month [22–24]. Thus, we cannot rule out the possibility that the high prevalence of insomnia among participants may have influenced on the prevalence of migraine and other headaches. Because of a very low participation rate and the high prevalence of insomnia among participants, generalization of our results to the entire population must accordingly be done with great caution.

## Conclusions

In this cross-sectional study evaluating 1-year prevalence of headache by a face-to-face interview, 43.1% had TTH, and 34.1% idiopathic stabbing headache. A total of 18.1% had active migraine (18.1%), whereas the lifetime prevalence of migraine was 32.8%.
